# Conserving the axilla in breast cancer

**DOI:** 10.3332/ecancer.2020.1090

**Published:** 2020-08-18

**Authors:** Felix Jozsa, Muneer Ahmed

**Affiliations:** Division of Surgery and Interventional Science, University College London, Royal Free Hospital, 9th Floor (East), Pond St, London NW3 2QG, UK; a https://orcid.org/0000-0001-8960-6049; b https://orcid.org/0000-0002-4504-1354

**Keywords:** breast cancer, axillary node metastasis, sentinel node biopsy, primary systemic therapy, axillary node clearance, radiotherapy

## Abstract

It is recognised that surgical conservatism is the most effective way of managing the axilla in breast cancer patients undergoing primary breast conserving surgery. The extended clinical scenarios in which a less aggressive approach can be safely adopted warrant consideration—including a group of patients who potentially could bypass surgical staging of the axilla altogether. The application of omission of further surgical management and axillary radiotherapy in the primary surgical and neoadjuvant chemotherapy settings are considered.

## Introduction

Surgical axillary nodal status remains relevant for the determination of adjuvant systemic therapies in breast cancer. Modern breast cancer care is based upon multimodal management and has since evolved several diagnostic and therapeutic techniques that allow for a more nuanced approach with the aim of avoiding complete axillary node clearance (ANC) where possible. This includes genomic assessments, such as MammaPrint [1], Oncotype dx [2] and Endopredict [3]. Patients may present with nodal disease detectable only on imaging or with clinically palpable disease. The advent of the use of primary systemic therapies (PST) adds further distinct patient groups to be considered. Here, we review the current evidence for surgical and non-surgical management strategies of axillary nodal metastases in breast cancer amongst all these patient subsets.

## Patients undergoing primary breast surgery

For patients presenting with primary invasive breast cancer, current practice with regards to the axilla includes a clinical and radiological assessment (most commonly ultrasound) at the time of work up [4]. The clinical assessment of the nodal status is vitally important as positive and negative groups represent distinct patient populations. The current standard of practice subdivides these two groups into further distinct groups based on the quantified metastatic axillary burden on staging (up to two nodes and greater than two nodes), however, this may not be as relevant as was once thought.

ANC was once a one-size-fits-all tool to enable locoregional control. The subsequent development of sentinel node biopsy (SNB) meant that the pathological status of some axillary nodes could be assessed prior to the completion of ANC. An important further step forward came from the results of the NSABP B-32 [5] and IBCSG 23-01 [6] trials which demonstrated that there was no statistical difference in overall survival (OS) between patients undergoing SNB alone versus SNB and ANC when the initial SNB was negative or positive for micrometastases, respectively. This began the debate regarding the threshold of nodal involvement at which SNB could be considered safe and sufficient management of the axilla. The value of being able to safely substitute SNB for ANC is based upon significantly improved functional arm outcomes, including reduced pain, numbness and swelling [7].

### Low and high burden at sentinel node biopsy

The landmark ACOSOG Z011 [8] trial designated a threshold of two or fewer macrometastatically involved nodes at SNB at which to observe the effect of withholding ANC. This trial enrolled patients with T1-2 primary breast cancers with no palpable lymphadenopathy undergoing primary breast surgery—in the form of breast conserving surgery and adjuvant radiotherapy and systemic therapy—and found no survival benefit for ANC over SNB alone. These results have shaped opinion and practice over the last decade [9] and in this patient group it is now widely accepted that completion ANC is unnecessary [10]—with SNB being considered as therapeutic for low nodal burden axillary disease. Further international clinical trials are underway to see if these results are fully replicable, and to answer some other pending issues raised by the Z11 trial. It is unclear, for example, what absolute value the threshold of two positive nodes has in determining the oncological risk to the patient: if three positive nodes are excised at SNB, could the patient avoid further axillary surgery? To answer this question, the INSEMA [11] trial will now adapt and expand on the Z11 protocol by including patients with up to three macrometastatically involved axillary lymph nodes at SNB. Furthermore, it will not include patients with axillary micrometastases (37.5% of patients included in Z11 had micrometastases only), helpfully consolidating the patient group being studied. An added consideration to the change of practice heralded after Z11 come from the results of the AMAROS [12] trial which indicate that in patients with a positive SNB, axillary radiotherapy provides equivalent locoregional control when compared with ANC and with less associated morbidity. Unsurprisingly, only 5% of patients in the AMAROS trial had three or more macrometastatically involved nodes at SNB—as would be expected in a clinically non-palpable axilla. These results should be interpreted in the context of applying axillary radiotherapy as an alternative to ANC in the—clinically negative, sentinel node positive—low burden axilla.

### Radiotherapy

Radiotherapy itself also has adverse side effects—including lymphoedema—and should be avoided where safely possible. In patients with early breast cancer and favourable biomarkers (using the composite IHC4+C score), the PRIMETIME [13] trial will investigate the ipsilateral breast disease recurrence rates when whole-breast radiotherapy is omitted following breast-conserving surgery and negative SNB. The role of axillary conservatism in low-risk groups will, therefore, need to be balanced and considered alongside the reductive use of radiotherapy. The PRIMETIME trial includes SNB, and the conservative omission of completion axillary node clearance relies upon the patient receiving whole-breast radiotherapy. Both strategies cannot be employed simultaneously and the effect of each strategy on recurrence and survival will, therefore, need to be untangled in future clinical trials.

### Preoperative ultrasound

The Z11 trial did not use axillary ultrasound to stratify patients prior to sentinel node biopsy. Since axillary ultrasound is routinely used is in clinical practice, the relevance of it on quantification of axillary burden is an important consideration.

In the case of a positive pre-operative AUS, the current standard is to either proceed with ANC or to commence upon PST to downstage the axilla. The practice of ‘fast-tracking’ positive AUS patients to ANC based on the assumption that, owing to its relatively low sensitivity [14], a positive AUS indicates high nodal burden, leads to gross overtreatment of this patient group. Up to 43% of patients with a positive pre-operative AUS who went on to have ANC had two or fewer involved nodes at surgery [15], meaning these patients could have been provided the same amount of oncological safety with a sentinel node biopsy. This process is complicated by the widespread use of core needle biopsy or fine-needle aspiration cytology (FNAC) to obtain pathology results pre-operatively. The pathological status of a single node biopsied following visualisation on AUS provides no valuable clinical information for the surgeon deciding how to manage the axilla [16], as it does not provide accurate information on the quantification of axillary burden—which requires sentinel node assessment. In view of this, an approach consisting of SNB enhanced by ‘clipping’ of the core-biopsy confirmed node—to ensure retrieval at surgery using a targeted axillary dissection (TAD)—in the primary surgical setting would provide this accurate staging assessment. This bespoke approach for primary surgery—supported by adjuvant axillary radiotherapy—is currently being explored within the TAXIS trial [17].

The current standard of practice consists of SNB being performed to confirm the node-negative status of the axilla—in the clinically and radiologically negative axilla. However, despite AUS’s relatively low specificity, its sensitivity is far more robust. Indeed, a pooled meta-analysis showed that among 5,139 patients with a negative axillary clinical and ultrasound examination pre-operatively, the negative predictive value of AUS was 95.1% for low nodal burden [16]. This supports that following a negative clinical and AUS pre-operatively, T1-2 breast cancer patients may be able to safely avoid surgical staging of the axilla altogether. Historical precedent for this approach comes from before the emergence of the concept of SNB, when various trials looked at ANC versus no axillary surgery in clinically node-negative patients, and these did not demonstrate a deleterious effect on overall or disease-free survival [11]. Furthermore, in a 7-year follow up of patients with a clinically negative axilla and a negative SNB there was a mere 1.7% incidence of axillary disease [18]. However, despite this data, the 2019 St. Gallen expert panel [19] recommends awaiting the full results of the SOUND [20] and INSEMA [11] clinical trials before changing practice to omit SNB in this patient group as both trials have arms that compare sentinel node biopsy with observation. Improving biomolecular marker assessment for identification of the 4% of patients with high nodal disease who present clinically and radiologically negatively is essential. For example, in patients with T1-2, N0, M0 disease and a negative SNB although the locoregional recurrence rates were very low as expected, the ACOSOG Z0010 study found higher rates in younger patients and those with hormone-receptor negative tumours [21].

### Patients undergoing mastectomy

Traditionally, patients undergoing mastectomy—regardless of their axillary node status—have been considered as a separate group in terms of the management of the axilla, owing to the omission of adjuvant radiotherapy in certain patients. Current indications for post-mastectomy radiotherapy (PMRT) include tumour size greater than 5 cm, T4 disease, 4 or more positive axillary lymph nodes and positive or close surgical margins [22]. All of the trials considering the benefit of omitting ANC in patients with low axillary node burden undergoing breast conserving surgery included adjuvant whole-breast radiotherapy as a part of protocol. This technique leads to some irradiation of the lower axillary inlet and may, therefore, contribute to locoregional control in that region. However, multiple studies comparing axillary disease recurrence between patients receiving whole-breast radiation alone and partial breast irradiation—in which, depending on the location of the tumour, the axilla would be far less likely to be irradiated—have shown no difference in axillary disease recurrence [11]. Equally, none of the trials completed to date looking at this issue have included patients undergoing mastectomy. This leaves an open question regarding the oncological feasibility of omitting ANC in some patients undergoing mastectomy. Axillary conservatism could subsequently lead to expansion of the indications for PMRT in order to avoid further surgical intervention to the axilla. The POSNOC [23] and SENOMAC [24] trials will include patients undergoing mastectomy and compare outcomes of axillary management versus adjuvant therapy alone in patients with two or fewer positive axillary nodes. These and future trials looking at including mastectomy cases in a Z11-type protocol, provided adjuvant radiotherapy is given will be key to defining the risk in this group.

## Patients undergoing PST

The use of neo-adjuvant chemotherapy (NACT) in breast cancer is successfully deployed in downsizing the primary tumour to permit breast conserving surgery, thus avoiding mastectomy. Long term survival outcomes in NACT versus adjuvant chemotherapy have been comparable [25]. In terms of the axilla, the patient’s nodal status can change pre- and post-NACT, and the value of SNB in this setting remains.

Three clinical trials have examined the false-negative rate (FNR) of SNB following NACT as previous retrospective series had demonstrated highly variable rates—some so high as to nullify the utility of the procedure in this setting [26]. ACOSOG Z1071 [27] reported an overall FNR of 12.6% in their patient cohort. Although unacceptably high for the predetermined 12% safety threshold, they found that when dual tracers were employed this reduced to 10.5% and, furthermore, that removing three or more nodes at SNB reduced the FNR to 9.1%. This was echoed in the SENTINA trial [28], which reported an overall FNR rate of 14.2%, which reduced to 7.3% when only patients in whom three or more nodes had been removed at SNB were considered. The SN FNAC trial [29] found comparable FNR rates of 11.5% when considering macrometastatic deposits as per the protocols for the Z1071 and SENTINA trials. However, when departing from those trial protocols, they found that mandatory use of immunohistochemistry (IHC) and defining metastases less than 0.2 mm as node-positive reduced the FNR to 8.4%.

In patients with node-positive disease prior to NACT, a biopsy can be performed to confirm pathological status of the node, and a radiological clip placed in the node. This is useful as the pathological response to NACT of the clipped node is used as a surrogate for the overall nodal status of the axilla. Indeed, a further finding from the Z1071 trial was that FNR of SNB in the 203 patients with a radiologically clipped node was 6.8% when the clipped node was in the SNB specimen [30]. So-called TAD, thus involves SNB with selective resection of the previously marked node using iodine-125 seed localisation placed 1–5 days before surgery [31]. The initial report of the use of this technique by Caudle *et al* [31] in 191 patients demonstrated an FNR of 2.0% for TAD versus 10.1% for SNB alone (although this improvement did not reach statistical significance). The marking axillary lymph nodes with radioactive iodine [32] procedure ( involves inserting radioactive seed at the time of biopsy (unlike TAD in which the seed is placed 1–5 days before surgery) without performance of SNB and reported a FNR of 7.0% in 100 patients and an identification rate of 97%.

These techniques seek to optimise the diagnostic and therapeutic value of an augmented sentinel node biopsy procedure to enable the minimum amount of surgical intervention in the axilla post-NACT. Removing the clipped node, and at least three nodes as a part of SNB is evidence-supported to provide good oncological control while avoiding ANC. If, at TAD, residual disease is found in the biopsy-proven node, then further axillary management is needed—current standard of practice would be to perform completion ANC. However, those with low axillary burden are likely being overtreated with this approach. These patients require assessment in future trials of no further management —in the context of breast conserving surgery post-NACT receiving whole breast radiotherapy—and also axillary radiotherapy. The ongoing Alliance A11202 trial [33] will assess whether axillary radiotherapy can provide equivalent locoregional control when compared to ANC in this patient group.

## Conclusions

The evidence-supported direction of axillary surgical management is of decreasing intervention to the axilla. This is supported in both the primary breast surgery and systemically-treated settings. The future refinements of surgery will be based upon the selection of omission of surgical staging, enhancing the bespoke nature of targeted axillary dissection, determination of the significance of residual disease—after PST—and further assessing what truly represents the threshold for low axillary burden. This will herald an exciting era of reduced morbidity for patients.

## Conflict of interest statement

The authors have no disclosures to make concerning financial or personal relationships that could inappropriately influence their work.

## Funding declaration

No funding was received for this study.
